# Editorial: Case Reports in Pediatric Cardiology: 2021

**DOI:** 10.3389/fcvm.2022.900584

**Published:** 2022-04-13

**Authors:** Inga Voges

**Affiliations:** Department of Congenital Heart Disease and Pediatric Cardiology, University Hospital Schleswig-Holstein, Kiel, Germany

**Keywords:** case report, May-Thurner syndrome, COVID-19, hypothyroidism, infective endocarditis, pericarditis, hypertrophic cardiomyopathy, long QT type 3

Case reports are of great interest for clinicians in the field of Pediatric Cardiology. One of the reasons is the rarity of many cardiac conditions in children that do not allow studies with large number of patients. In addition, the clinical information and experience in case reports might help treating other pediatric patients in future.

A good case report carries this information in that way that readers can learn from it and ideally learning points are provided. In this Research Topic selected pediatric case reports published in 2021 are presented and illustrate that case reports particularly contribute to the management of rare diseases. This is nicely demonstrated by Ji et al. who report a case of May-Thurner syndrome in a pediatric patient. A syndrome that normally and typically affects women in their third to fifth decade of life (1, 2). Apart from describing the diagnostic methods, the authors also discuss potential treatment options. However, case reports are particularly useful if they cover cutting-edge topics such as this was shown by Bigdelian et al. They present a case series (three cases) of patients with COVID-19 infection and an intracardiac thrombosis that was successfully treated by cardiac surgery. Although, children are known to be typically less affected by COVID-19, this report demonstrated that physicians should also be aware of a potential hypercoagulopathy in young patients. That we also must think about other causes than COVID-19 when children present with fever at the present time, is nicely described by Lubocka and Sabiniewicz who present a child with fibrinous pericarditis. The various infective and non-infective etiologies of pericarditis are discussed by the authors, and treatment strategies are presented. In the patient described a chest trauma was considered as a possible reason for the pericarditis. Sometimes, other diseases can mimic cardiac inflammation, and this was shown by Zhang et al. who report a 6-year-old boy with frequent convulsions and loss of consciousness who was initially diagnosed with fulminant myocarditis. Electrocardiogram at presentation showed a third-degree atrioventricular block and echocardiography diagnosed an enlarged left ventricle. The child was also found to have a developmental delay. A review of the patients' history finally brought clarity about the diagnosis. The patient suffered from hypothyroidism and in the discussion the effects of hypothyroidism on the cardiovascular system are described (Zhang et al.). Infective endocarditis is another rare condition in children, especially if they do not suffer from congenital heart disease. The case report by Joye et al. is presenting a 6-year-old girl with infective endocarditis due to an infection with *Kingella kingae*. Rapid cardiac deterioration required emergency venoarterial extracorporeal membrane oxygenation (Joye et al.). Beside vegetations at the aortic valve level, a para-aortic abscess was diagnosed, and the patient underwent surgical treatment. A second surgery was necessary 3 months later due to a para-aortic pseudoaneurysm with good results. The authors discuss the literature about *K. kingae* infective endocarditis and their treatment strategy.

Increasing interest is paid to inherited cardiac conditions in pediatric patients. In this Research Topic two final articles cover this aspect and show the importance of genetic testing (Bagkaki et al.; Li et al.). Bagkaki et al. report about a family with long QT type 3 due to a missense mutation in the *SCN5A* gene. They show that the index patient, a neonate, was successfully treated with an oral medication of propranolol and mexiletine. Mexiletine was able to normalize the prolonged QTc at low therapeutic serum levels (Bagkaki et al.). In the discussion section, the effects of mexiletine and the challenge of mexiletine treatment in very young children are discussed (Bagkaki et al.). The second case report by Li et al. describes a 7-year-old girl with hypertrophic cardiomyopathy and previous history of recurrent cardiac arrests. Whole exome sequencing was performed showing a *de novo* c.2723T>C (p.L908P) heterozygous mutation in the *MYH7* gene. The patient underwent implantation of an implantable cardioverter defibrillator and received antiarrhythmic and heart failure treatment (Li et al.).

Each article of the presented selection of case reports fulfills the criteria of a “good case report.” The reports often go beyond describing and discussing the particular case but even discuss the presented topic in detail and hereby provide a mini review.

The collection also shows the increasing knowledge in pediatric cardiology and encourages other colleagues for presenting their interesting cases and for performing research studies on rare pediatric cardiac diseases.

## Author Contributions

The author confirms being the sole contributor of this work and has approved it for publication.

## Conflict of Interest

The author declares that the research was conducted in the absence of any commercial or financial relationships that could be construed as a potential conflict of interest.

## Publisher's Note

All claims expressed in this article are solely those of the authors and do not necessarily represent those of their affiliated organizations, or those of the publisher, the editors and the reviewers. Any product that may be evaluated in this article, or claim that may be made by its manufacturer, is not guaranteed or endorsed by the publisher.
